# Developmental Trajectories in Spatial Visualization and Mental Rotation in Individuals with Down Syndrome

**DOI:** 10.3390/brainsci11050610

**Published:** 2021-05-10

**Authors:** Elizabeth Maria Doerr, Barbara Carretti, Enrico Toffalini, Silvia Lanfranchi, Chiara Meneghetti

**Affiliations:** 1 Department of General Psychology, University of Padova, 35131 Padova, Italy; barbara.carretti@unipd.it (B.C.); enrico.toffalini@unipd.it (E.T.); chiara.meneghetti@unipd.it (C.M.); 2 Department of Developmental Psychology and Socialization, University of Padova, 35131 Padova, Italy; silvia.lanfranchi@unipd.it

**Keywords:** developmental trajectories, spatial visualization, mental rotation, individuals with Down syndrome

## Abstract

Background. The analysis of developmental trajectories of visuospatial abilities in individuals with Down Syndrome (DS) remains an unexplored field of investigation to examine in depth. The study aimed to fill such a gap by examining changes in two visuospatial abilities: spatial visualization (the ability to manage spatial stimuli) and mental rotation (the ability to rotate spatial stimuli). Method. Eighty-seven participants with DS, aged between 7 and 53 years (forty-seven males and forty females), completed spatial visualization and mental rotation tasks. Changes in these two abilities were analyzed in relation to chronological age and developmental level, the latter derived from Raven’s Colored Progressive Matrices. Results. Chronological age was linearly associated with spatial visualization performance, whereas mental rotation performance increased until 14 years of age and then decreased. Developmental level was linearly associated with increased performance in spatial visualization, the trend in mental rotation was segmented with an increase after 5 years of age. Furthermore, developmental trajectories in mental rotation depended on the rotation degree. Conclusion. Chronological age explains a modest quote of variance. Developmental level better describes changes in spatial visualization and mental rotation of individuals with DS.

## 1. Introduction

Visuospatial abilities allow the generation, retention, and transformation of abstract visual images [1], which are the basis of spatial thinking [2]. Visuospatial abilities are classically distinguished into three sub-factors [3]: spatial perception (the ability to locate an object, analyze the relation between spatial elements), spatial visualization (the ability to perform multi-step manipulations of complex spatial information), mental rotation (the ability to manipulate figures by rotating 2D or 3D stimuli). Such skills, and in particular spatial visualization and mental rotation, are mostly examined in the domain of typical development (TD) in relation to several everyday tasks, such as environment learning (like navigation or map learning, [4]) and school learning (like math, [5]).

Examining the impact of visuospatial abilities in Down syndrome (DS) could be a compelling topic, considering the constraints imposed by the syndrome. DS is a genetic syndrome caused by chromosome 21 trisomy with an intelligence quotient between 25 and 70, and a mental age between 5 and 6 years [6,7]. Individuals with DS are generally recognized as weaker in visuospatial abilities and stronger in verbal ones [6,8]. This is still an overgeneralization due to the multiple sets of visuospatial skills [9]. For this reason, visuospatial abilities in individuals with DS deserve to be studied in depth. 

An effective modality that has recently been suggested to examine individual and developmental differences consists in analyzing the development of a certain ability across age by identifying its maximum level of development and its decrease. This approach in the Atypical Developmental (AD) population results to be particularly informative to understand how abilities develop according to chronological age and cognitive functioning: the level of the latter can vary within this population despite the chronological age [10]. 

The present study examined the developmental trajectories of visuospatial skills by taking into account spatial visualization and mental rotation in individuals with DS across different chronological ages and levels of cognitive development.

### 1.1. Visuospatial Abilities in Down Syndrome

Previous studies examining visuospatial abilities in individuals with DS frequently made group comparisons with TD children (matched for cognitive functioning level using verbal or visuospatial tasks) while others compared the group with DS to other AD populations or peers of the same chronological age. Most of the studies focused on the assessment of visuospatial working memory (VSWM), which concerns the retention and processing of visuospatial information. Given that VSWM can be differentiated in further sub-components [11], some studies analyzed visual and spatial aspects of WM in individuals with DS in depth, showing a non-homogeneous profile. Indeed, various findings suggested that spatial components are in line with developmental levels, whereas visual aspects are lower than those to be expected [12,13,14]. A further distinction was made within spatial WM between a spatial simultaneous and spatial sequential component. Previous studies suggested that spatial-sequential WM is consistent with developmental levels in individuals with DS, as opposed to a relative impairment in spatial-simultaneous WM [15,16,17,18]. 

Other evidence concerns complex visuospatial abilities. Yang et al. [9] examined the results in the literature regarding abilities of spatial visualization (named visual-construction abilities in their review) and mental rotation (as well as closure and navigation abilities), which were examined in the present study.

Spatial visualization consists in managing spatial stimuli as mentally complete or assembling figures. An example of task assessing spatial visualization is the Block design task, as it requires the reconstruction of a geometric configuration, introduced with an image, and its reproduction using blocks (WISC-IV, [19]; WAIS–IV, [20]); or the Spatial relations subtest taken from the Primary Mental Ability batteries (e.g.**,** PMA-K1, [21]) in which participants are required to choose the piece that should complete the target figure, among four options. Yang et al. [9] illustrated a summary of the performance of participants with DS in Block design and other tasks, comparing their results with those in TD and other AD populations, such as Williams syndrome (WS), X fragile syndrome and intellectual disability. Thus, TD participants were better in the Block design task, whereas participants with DS and WS demonstrated having similar levels, just like participants with intellectual disability and X fragile syndrome. Interestingly, individuals with DS tended to correctly complete the outer configuration of the items of the Block design task, while individuals with WS were more efficient in the construction of the inner configuration [22]. Participants with DS were instead the most efficient in drawing tasks. Moreover, participants with DS obtained higher standard scores in task assessing visuo-motor integration (VMI) compared to their IQ level, contrarily to people with other intellectual disabilities who reported equal levels of IQ and VMI, or people with WS reporting a higher IQ level [23]. The results gathered from Yang et al. [9] seem to confirm the presence of strengths and weaknesses in spatial visualization tasks related to the type of requests.

Mental rotation (MR) is the ability to turn 2D and 3D objects mentally [24,25,26] and it is scarcely examined in DS. Studies considering the MR ability in individuals with DS required participants to compare couples of stimuli (like letters, [24]), or drawings [25,26] in different degrees of rotation ranging from 0° to 180°. Being able to recognize identical stimuli with a wide rotation angle is an indicator of developed mental rotation skills. Hinnell and Virji-Babul [24] asked participants to identify whether couples of letters were reversed or not by presenting them in different degrees of rotation (0°, 45°, 90°, 135°, and 180°). The results showed that the DS and TD groups (matched for receptive vocabulary) did not significantly differ in response times, but they did in accuracy, with the group of individuals with DS performing more poorly compared to TD children, whose performance was nearing to a ceiling effect. Moreover, the performance of individuals with DS dropped to chance level when considering the maximum degree of rotation. Vicari et al. [25] used L- or S-shaped sticks with full or empty circles at the two ends (rotated between 45° and 270°); and geometrical drawings created ad hoc. In both tasks, individuals with DS and TD (matched for verbal and visuospatial abilities) were shown several options and asked to choose the one matching the target stimulus. Results showed no effect of group on total accuracy, but the reported mean scores revealed a floor effect in both groups. However, the authors did not analyze performance as a function of degree of rotation, which is also informative of the development of this ability in early age [27,28]. More recently, Meneghetti et al. [26] assessed mental rotation with the Ghost Picture Test (GPT; adapted by Frick et al. [27]) which consists in couples of ghost figures that need to be compared and judged as identical, while assuming different degrees of rotation (0°, 45°, 90°, 135°, and 180°), or specular positions. Results showed that individuals with DS were less accurate in mental rotation than TD children (matched for receptive vocabulary), with larger differences between groups for smaller angles of rotation (0–45°); individuals with DS could not mentally rotate through 180°, while TD children could. 

Overall these results showed that individuals with DS are able to rotate correctly until a certain extent, and the degree of rotation appears to be important to take into account. 

### 1.2. Visuospatial Abilities, Developmental Trajectories, and Gender Differences in TD Children

Some studies analyzed developmental trajectories in TD children, an example being a longitudinal study regarding visuospatial skills in elementary school children (7–9 age range, [29]). Visuospatial skills were measured with the two-dimensional mental rotation version of the PMA subtest [30] and a spatial relations task [31] requiring two images, among other options, which could match the target image by rotating together. Overall the results suggested that the developmental trajectory was better described by a linear increase across ages. Moreover, the authors considered the effect of the performance level (based on high and low visuospatial tasks performance), gender, verbal working memory and socioeconomic status. Students with low visuospatial skills were characterized by lower working memory, lower socioeconomic status and were most likely to be female, despite the absence of significant gender differences in general. Therefore, stronger effects of gender emerged only among participants who reported lower visuospatial skills. Compelling findings were described in another longitudinal study [32], focusing on TD students from elementary school (7–9 age range), which analyzed developmental trajectories in spatial visualization (Puzzle task, [33]) and spatial perception (Water-level task, [3,34]) tasks. A linear increase of all skills across the years was reported. It should be noted that an influence of spatial skills and math performance was found. Further performance differences were found across gender and school grade, with a better performance in males and with an increase in age. Consequently, the choice of the spatial task could be crucial due to the fact that each skill could bring different developmental trajectories. Harris et al. [35] studied the effects of a measure for spatial visualization, such as mental folding (which requires to imagine folds applied to a piece of paper), in young children from four to seven years of age. The obtained results were in line with previous findings, with mental folding appearing at age five. No differences based on gender were found. 

Visuospatial abilities, such as mental rotation, are related to frames of reference. The frame of reference is distinguished in object to object relation (allocentric) or subject to object relation (egocentric) and represents the modality to encode and memorize information; moreover frames develop across age [36]. For instance, Nardini et al. [37] showed that when children, aged from 3 to 6 years, were supposed to retrieve hidden toys as a task, they obtained benefits from spatial representations that were not purely egocentric, i.e.**,** from their own point of view. Viewpoint independent recall, which is based on an object-referenced view considering the array and bordering landmarks, emerged at age five (for a review see [38]). The results confirmed the presence of basic components of spatial competence already starting at age three, which are gradually supplemented by object-referenced representations that are related to visuospatial performance (as based on mental rotation and spatial visualization) throughout the following years until adult life.

Another topic of interest in the literature on visuospatial abilities is the role of gender in explaining differences in performance, which influence spatial thinking [39]. In a meta-analysis, Lauer and Yhang [40] analyzed the effect sizes of children and adolescents in mental rotation and highlighted the presence of a small advantage in males in rotation during childhood, which increased to a moderate effect in adolescence. Afterwards, gender differences remained stable in adult life. [41]. Nonetheless, the role of gender as a predictor of visuospatial skills remains undefined. In fact, gender differences could be reduced in relation to several factors (such as high socioeconomic status [42]) and related to the task, as the type of objects, with weaker gender differences when no resemblance to the cube configuration is used [43], time of exposure [44], or in a group testing context, rather than an individual one [41]. 

### 1.3. Visuospatial Abilities and Developmental Trajectories in Individuals with DS

The developmental trajectories approach has already been employed to explore the development of individuals with DS in several domains such as reading [45], general cognitive, language, motor, and socio-emotional development [46]. Studies seldomly examined developmental trajectories in the visuospatial domain. For example, Carretti et al. [47] analyzed developmental trajectories in spatial sequential and spatial simultaneous WM tasks. The trajectories of chronological age (age range: 7–53 years) were better described through a segmented model [48] showing increased performance until approximately 13 years of age, followed by a rather flat progress. In contrast, the increase in performance throughout the developmental level (age range: 2–9 years), considered with both visuospatial and verbal-measures, such as Raven’s Colored Progressive Matrices (CPM, Italian adaptation [49]) and Peabody Picture Vocabulary Test revised (PPVT-R [50]) respectively was linear, without significant differences between the type of measure used (verbal vs. spatial) to estimate the developmental level. These results apparently contrasted with those of Carney et al. [51] who analyzed the developmental trajectories of verbal and spatial WM in individuals with DS, reporting a linear increase with chronological age (age range: 10–21 years) and mental age (age range: 4–7 years; assessed with the Stanford–Binet Abbreviated Battery, ABIQ, [52]). Carney et al. [51] found no differences in the increase slope between the two WM components. Divergent results between the two studies may simply be due to the differences in the range of ages which were larger in the study of Carretti et al. [47] compared to that of Carney et al. [51]. In addition, only the linear term was tested in the latter study. 

Purser et al. [53] compared developmental trajectories across TD, DS, and WS in large-scale visuospatial abilities (i.e., navigation within an environment, recall of paths, landmarks, or shortcuts), considering the developmental level instead of chronological age, since the latter was not associated with visuospatial performance. In particular, participants were instructed to learn and recall routes and landmarks within a virtual environment. The developmental level was calculated with a non-verbal measure (CPM, [54]). The developmental trajectories analyzed the relationship between accuracy in route recall and CPM scoring to verify whether route-learning skills developed at different rates in each group. Results highlighted a general difficulty in participants with DS in route recall compared to TD and WS groups who committed fewer recall mistakes. The trajectories demonstrated a significant relation between CPM and route-learning performance with a linear trend; indeed, higher CPM scores were associated with superior performance. However, low CPM scoring had a more negative impact on performance, whereas participants with DS who obtained high CPM scoring approached similar levels to the TD group. Overall, participants with DS demonstrated an efficient use of landmarks to recall routes, despite a more significant amount of errors compared to the other groups. Purser et al. [53] showed that non-verbal reasoning level is informative in explaining performance in a visuospatial task.

The results of some studies examining the developmental trajectories in individuals with DS seem to encourage the use of trajectories with the purpose of studying the evolution of specific skills in time although such methodology is less frequent in studies that are more focused on AD. For these reasons, developmental trajectories were also hypothesized in this study to analyze changes in two visuospatial abilities.

### 1.4. Rationale and Aim of the Study

Overall the results show that developmental trajectories in TD children are linear, considering both spatial visualization and mental rotation [29,32,35]. Instead, fewer studies have analyzed developmental trajectories in individuals with DS and in visuospatial abilities in particular. The present study aimed to examine developmental trajectories in visuospatial abilities in individuals with DS, focusing on spatial visualization and mental rotation. 

Therefore, the objectives of the study were: to analyze developmental trajectories in two measures of visuospatial abilities, i.e., spatial visualization and mental rotation, based on chronological age and mental age, and controlling for the role of gender. Furthermore, specific developmental trajectories were considered for mental rotation, distinguishing between the angles of rotation 

In line with available previous literature on children with TD [29,32,35] and on individuals with DS [47,51,53], it has been hypothesized that both chronological age and developmental level could explain the performance of visuospatial tasks. A different trend depending on chronological age or developmental level was hypothesized. In particular, the evidence of linear [51] vs. segmented trajectories [47] were tested, while exploring the possibility of trajectories changing in function of the type of considered visuospatial ability. Finally, developmental trajectories were analyzed (in the case of mental rotation) as a function of the degree of rotation. 

Since gender differences in visuospatial performance are reported in the literature [41] also at an early age [40], gender was included as a covariate.

## 2. Materials and Methods

### 2.1. Participants

The present study was approved by the Ethics Committee for Research in Psychology of the University of Padova. Eighty-eight individuals with DS partook in the study, however an outlier was identified and subsequently dropped from the dataset. Therefore, the total sample consisted of eighty-seven individuals with DS (forty females; M age = 19.46 years; SD = 9.57; age range = 7.75–53.25 years). All participants were selected from previous studies that focused on other aspects, such as working memory or recall and map drawing: twenty-nine partook in a research on path recall [55], twenty-nine took part in a study concerning visuospatial descriptions and map drawing [56], whereas twenty-nine were included in a study focused on spatial cognition and navigation [57].

### 2.2. Materials

#### 2.2.1. Visuospatial Reasoning Task. Raven’s Colored Progressive Matrices (CPM)

The CPM (original [54], Italian Adaptation by Belacchi et al. [49]) consists of thirty-six increasingly complex colored matrices, where each matrix has a missing piece: the respondent is asked to choose the best fit for the missing piece among six options. The reliability is good: the test–retest stability and convergent validity with other intelligence tests is strong in all international versions of the CPM, with r in 0.60–0.90 [49]. The final score is the number of correctly completed matrices (ranging between 0 and 36).

#### 2.2.2. Mental Rotation Task. Ghost Picture Test (GPT)

The task (adapted by Frick et al., [27]) consists of twenty-one items, each depicting the silhouette of a ghost inside a circle, at the top of a page. Two similar silhouettes are presented beneath the target figure, but only one of them is identical to the target but is rotated, while the other is a mirror image of the target figure. Participants are asked to choose the figure that matches the target silhouette. Six practice items are administered before the task and, for the first of these, participants manually rotate transparent paper cutouts of the two figures to choose from. The items used in the task differ in terms of the angle of rotation of the figure corresponding to the target ghost, which could be 0° (three items), 45° (three items), 90° (seven items), 135° (four items), and 180° (four items). The score is obtained from the total number of correct answers divided by the angle of rotation of the correctly identified figures. A total score of all correct responses is also calculated. Note that, when the GPT is examined as the response variable in the subsequent analysis, dichotomous responses (“right”/“wrong”) are used for single items to obtain better estimates of the models parameters. The chance level corresponds to half of the answers being correct (for a total score of 10.5). No reliability measure was calculated since this is a performance task that involves dichotomous responses.

#### 2.2.3. Spatial Visualization Task. Primary Mental Abilities–Spatial Relations–K1 (PMA-K1)

This test [21] consists of twelve items, each showing an incomplete target figure and four different pieces, to reconstruct a complete figure. Participants are asked to choose the piece that completes the target figure. The internal consistency is good (adjusted split-half correlation r = 0.81 in preschoolers). The final score is the total number of correct responses, ranging between 0 and 12 (3 being the chance level).

### 2.3. Procedure

Parents (or legal representatives) and individuals with DS over 18 years of age offered their informed consent. Afterwards, participants were tested individually in a single session lasting 30 min at the day center or school, where a table and chairs were available. In all studies, the CPM was administered first, followed in a counter-balanced order by GPT and PMA-K1, across participants. Specific instructions were given for each measure, and the participants practiced with examples before approaching the tasks to assure they understood.

## 3. Results

The data analysis was conducted with the R software [58].

### 3.1. Descriptive Statics

According to the age-equivalent scoring of the CPM, the mean visuospatial mental age was M = 5.62, SD = 1.48. Table 1 illustrates the total scores obtained in three visuospatial tasks. 

### 3.2. Analysis of the Developmental Trajectories

Performance was evaluated as a function of age-equivalent and chronological age values through linear and segmented regression models due to the main interest in using a simpler model that could better explain such effects without the risk of overfitting data. The “segmented” library [59] of R was applied to fit those models.

For GPT, proportioned scores were considered when total scores for each rotation angle were considered, due to the unequal number of items for each rotation angle. Generally, participants gradually struggled with the increase of rotation; in fact, the total proportioned scores for each angle were: GTP 0° (M = 0.75, SD = 0.27), GTP 45° (M = 0.63, SD = 0.30), GTP 90° (M = 0.63, SD = 0.23), GTP 135° (M = 0.57, SD = 0.27), and GTP 180° (M = 0.50, SD = 0.26).

All regression models were tested for a null, a linear, and nonlinear (segmented) trajectory. For any purpose of model selection, the best fitting model was chosen with the Akaike Information Criterion (AIC, lower is better; [60]) and significant *p*-value between models (*p* < 0.05). Whenever the segmented model demonstrated higher AIC or *p* ≥ 0.05, the simpler model, hence the linear one, was kept.

Firstly, spatial visualization was considered, with the total score of PMA-K1 as dependent variable, gender as covariate and age as predictor (either chronological, or developmental level calculated with the CPM age-equivalent scoring). Secondly, mental rotation (GPT) was considered in terms of:

- total raw score as dependent variable, gender as covariate and age as predictor (either chronological or developmental level);
- each rotation degree. Therefore, the dependent variable was the total score for the specific rotation (0–45–90–135–180°) and the predictor was age once again, either chronological or developmental level.

#### 3.2.1. Spatial Visualization

**Figure 1:**
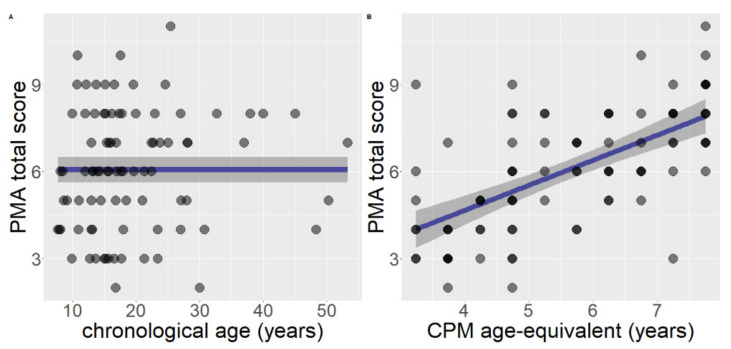
The representation of regressions for spatial visualization (PMA total raw score), including gender as covariate (**A**) model with chronological age as predictor; (**B**) model with developmental level, measured with age-equivalent scoring, as predictor.

When considering chronological age as predictor and gender as covariate on the total PMA–K1 score, the segmented model (AIC = 377.76) was not significantly better than the linear model (AIC = 376.27), F (2, 82) = 1.20, *p* = 0.305, and the latter was not significantly better than the null model (AIC = 374.61), F (1, 84) = 0.32, *p* = 0.570. Therefore, the null model was retained as the best one (Figure 1A).

When considering developmental level as predictor and gender as covariate on the total PMA–K1 score, the iterative procedure of the segmented model did not find any breakpoint on the linear relationship. The linear model (AIC = 332.32) significantly outperformed the null model (AIC = 374.60), F (1, 84) = 55.75, *p* < 0.001. Therefore, the linear model was retained as the best one. The estimated relationship was B = 0.87 (95% CI: 0.64, 1.10), SE = 0.12, *p* < 0.001. R^2^ = 0.43 (Figure 1B).

#### 3.2.2. Mental Rotation

**Figure 2:**
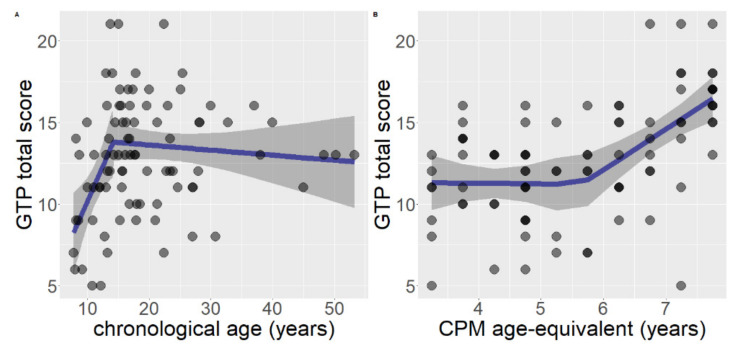
Segmented regressions for mental rotation (GTP total raw score), considering gender as covariate (**A**) model with chronological age as predictor; (**B**) model with developmental level measured with age-equivalent scoring, as predictor.

In the case of GTP total raw score, chronological age as predictor and gender as covariate, the segmented model (AIC = 461.36) was both significantly better than the linear model (AIC = 471.61), F (2, 82) = 7.30, *p* = 0.001, and significantly better than the null model (AIC = 471.30), F (3, 82) = 5.49, *p* = 0.002. Therefore, the segmented model was retained as the best one. The estimated breakpoint was at age = 14.13 years (95% CI: 11.40, 16.86). The overall segmented model had R^2^ = 0.17. The estimated relationship before the breakpoint was B = 0.87, SE = 0.36, *p* = 0.025, R^2^ = 0.19. The estimated relationship after the breakpoint was B = −0.03, SE = 0.04, *p* = 0.431, R^2^ = 0.01 (Figure 2A).

When considering developmental level on the total GTP score, with gender as covariate, the segmented model (AIC = 444.13) was both significantly better than the linear model (AIC = 447.70), F (2, 82) = 3.73, *p* = 0.028 and significantly better than the null model (AIC = 471.40), F (3, 82) = 12.68, *p* < 0.001. Consequently, the segmented model was retained as the best one. The estimated breakpoint was at 5.65 years of age (95% CI: 4.55, 6.75). The overall segmented model had R^2^ = 32. The estimated relationship before the breakpoint was B = −0.008, SE = 0.66, *p* = 0.989, R^2^ = 0.01. The estimated relationship after the breakpoint was B = 2.40, SE = 0.68, *p* = 0.001, R^2^ = 0.26 (Figure 2B).

**Figure 3:**
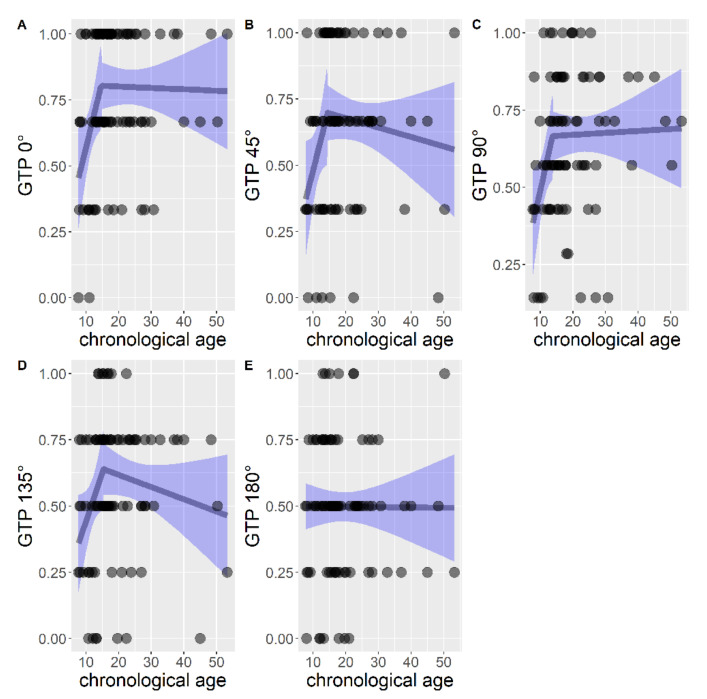
Regression models for each rotation level, with chronological age as predictor: (**A**) segmented model for rotation at 0°; (**B**) segmented model for rotation at 45°; (**C**) segmented model for rotation at 90°; (**D**) segmented model for rotation at 135°; (**E**) null model for rotation at 180°.

A group of regressions was considered with the purpose of comparing and choosing the type of model that could better explain the effect of chronological age on rotation performance, considering each rotation angle (Figure 3).

The preferred model for GTP 0° was the segmented (AIC = 15.21), which fitted better than the linear model (AIC = 20.42), F (2, 83) = 4.64, *p* = 0.012 and better than the null model (AIC = 21.30) F (3, 83) = 4.12, *p* = 0.009. Therefore the segmented model was ultimately kept. The estimated breakpoint was at age 14.64 (95% CI: 10.90, 18.38). The overall segmented model had R^2^ = 13. The estimated relationship before the breakpoint was B = 0.05, SE = 0.03, *p* = 0.068, R^2^ = 0.12. The estimated relationship after the breakpoint was B = −0.0005, SE = 0.003, *p* = 0. 871, R^2^ = 0.0005 (Figure 3A).

The preferred model for GTP 45° was the segmented (AIC = 40.76), which fitted better than the linear model (AIC = 43.57), F (2, 83) = 3.38, *p* = 0.039, but not significantly better than the null model (AIC = 41.87), F (3, 83) = 2.35, *p* = 0.078. Nonetheless, the segmented model was chosen. The estimated breakpoint was at age 14.50 (95% CI: 10.14, 18.85). The overall segmented model had R^2^ = 0.08. The estimated relationship before the breakpoint was B = 0.05, SE = 0.03, *p* = 0.108, R^2^ = 0.10. The estimated relationship after the breakpoint was B = −0.003, SE = 0.004, *p* = 0.452, R^2^ = 0.01 (Figure 3B).

The segmented model (AIC = −6.03) for GTP 90° fitted better than the linear model (linear AIC = −2.82), F (2, 83) = 3.59, *p* = 0.032 and better than the null model (AIC = −1.84), F (3,83) = 3.44, *p* = 0.020. Thus, the segmented model was chosen. The estimated breakpoint was at age 13.75 (95% CI: 10.19, 17.31). The overall segmented model had R^2^ = 0.11. The estimated relationship before the breakpoint was B = 0.04, SE = 0.02, *p* = 0.098, R^2^ = 0.11. The estimated relationship after the breakpoint was B = 0.0002, SE = 0.003, *p* = 0.932, R^2^ = 0.0001 (Figure 3C).

The model chosen for GTP 135° was the segmented one (AIC = 19.65), which fitted better than the linear model (AIC = 22.78), F (2, 83) = 3.54, *p* = 0.033, but not significantly better than the null model (AIC = 3, 83) = 2.39, *p* = 0.075. Nonetheless, the segmented model was chosen. The estimated breakpoint was at age 15.42 (95% CI: 10.91, 19.92). The overall segmented model had R^2^ = 0.08. The estimated relationship before the breakpoint was B = 0.03, SE = 0.02, *p* = 0.095, R^2^ = 0.08. The estimated relationship after the breakpoint was B = −0.005, SE = 0.003, *p* = 0.199, R^2^ = 0.03 (Figure 3D).

The segmented model in GTP 180° (AIC = 18.89) was not significantly better than the linear model (AIC = 17.47), F (2, 83) = 1.25, *p* = 0.292 and the latter was not significantly better than the null model (AIC = 15.47), F (1, 85) = 0.002, *p* = 0.965. Therefore the null model was considered the best (Figure 3E).

**Figure 4:**
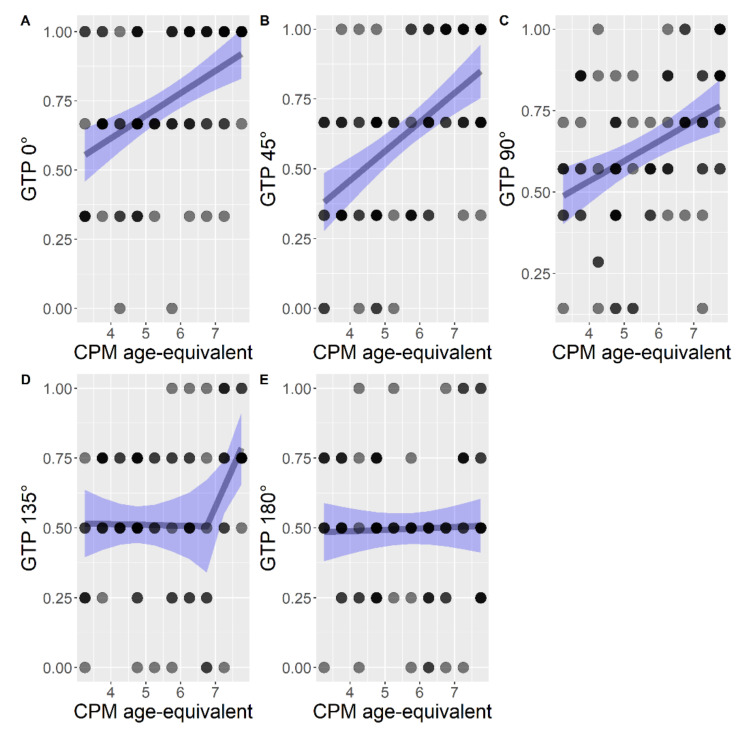
Regression models for each rotation level, with developmental level as predictor: (**A**) linear model for rotation at 0°; (**B**) linear model for rotation at 45°; (**C**) linear model for rotation at 90°; (**D**) segmented model for rotation at 135°; (**E**) null model for rotation at 180°.

A series of regression models were compared and chosen to better explain the relation between the developmental level (calculated with age-equivalent scoring) and performance in items with a specific rotation angle. The best fitting model was chosen to explain developmental level in each rotation angle (Figure 4).

In GTP 0° the linear model (AIC = 3.79) fitted better than the null model (AIC = 21.30), F (1, 85) = 21.36, *p* < 0.001, but not significantly better than the segmented model (AIC = 6.14) F (2, 83) = 0.80, *p* = 0.454. Nonetheless, the linear model was kept with the purpose of avoiding overfitting. The estimated relationship was B = 0.08 (95% CI: 0.05, 0.12), SE = 0.02, *p* < 0.001, R^2^ = 0.20 (Figure 4A).

The linear model (AIC = 17.53) explained data in GTP 45° better than the null model (AIC = 41.87), F (1, 85) = 30.05, *p* < 0.001, but not significantly better than the segmented model (AIC = 20.41), F (2, 83) = 0.540, *p* = 0.584. Nonetheless, the linear model was kept. The estimated relationship was B = 0.10 (95% CI: 0.07, 0.14), SE = 0.02, *p* < 0.001, R^2^ = 0.26 (Figure 4B).

The linear model explained data in 90° GTP better than the null model (AIC = −1.84), F (1, 85) = 14.93, *p* < 0.001, but not significantly better than the segmented model (AIC = 2, 83) = 2.62, *p* = 0.079. The linear model was kept. The estimated relationship was B = 0.06 (95% CI: 0.03, 0.09), SE = 0.01, *p* = 0.0002, R^2^ = 0.15 (Figure 4C).

The segmented model for GTP 135° (AIC = 13.43) fitted better than the linear model (AIC = 16.10), F (2, 83) = 3.31, *p* = 0.041 and better than the null model (AIC = 20.85), F (3, 83) = 4.61, *p* = 0.005. Thus, the segmented model was chosen. The estimated breakpoint was at age 6.75 (95% CI: 5.40, 8.10). The overall segmented model had R^2^ = 0.14. The estimated relationship before the breakpoint was B = 0.01, SE = 0.03, *p* = 0.582, R^2^ = 0.005. The estimated relationship after the breakpoint was B = 0.35, SE = 0.12, *p* = 0.008, R^2^ = 0.23 (Figure 4D).

The segmented model in GTP 180° (AIC = 16.05) was not significantly better than the linear model (AIC = 17.40), F (2, 83) = 2.63, *p* = 0.078 and the latter was not significantly better than the null model (AIC = 15.47), F (1, 85) = 0.07, *p* = 0.785. Therefore the null model was considered the best (Figure 4E).

## 4. Discussion

The aim of the present study was to analyze the developmental trajectories of two aspects of visuospatial abilities, i.e., spatial visualization and mental rotation. The developmental trajectories were considered within a range of participants with DS aged between 7 and 53 years with a cross-sectional design. In view of their relevance for everyday functioning, there is indeed growing interest regarding visuospatial abilities in individuals with DS [9]; however, few studies have tackled the issue with this methodological approach, which allows the understanding of how cognitive abilities develop based on chronological age and cognitive maturation [10]. 

In particular, in the present study regression analyses were used to explore the influence of both chronological age and visuospatial reasoning developmental level, measured with the CPM age-equivalent scoring, on spatial visualization and mental rotation performance. In the latter case, the difficulty of rotation, operationalized in terms of rotation angle, was also taken into account. Gender was inserted into the models as covariate given that visuospatial performance is usually influenced by gender [41].

Overall, results showed that both chronological age and developmental level explained a portion of variance in both types of visuospatial tasks. However, the developmental trajectories varied in spatial visualization (PMA–K1) and mental rotation (GTP) tasks. 

The regressions considering performance in the PMA-K1 task as dependent variable showed evidence in favor of linear models, especially when the developmental level was inserted as the predictor. The linear model better represented the trajectory of spatial visualization development in comparison with the segmented model (using AIC as comparison). No significant effect of gender was found. It should be noticed that the variance explained when the developmental level was considered as the predictor is higher (43%) in comparison with chronological age which was better described by a null model, characterized by a flat curve. This clearly highlights the gap between chronological age and developmental level which characterize individuals with intellectual disabilities: as opposed to typical development, the increase in chronological age does not correspond to a cognitive maturation of visuospatial abilities [51,53]. The pattern of results, with a larger portion of variance explained by the developmental level compared to chronological age, confirmed this asynchrony. 

Furthermore, in the case of GPT, regression analyses showed that both chronological age and developmental level contributed to performance. However, as opposed to PMA-K1, the segmented model in this task better represented the developmental trajectory. In particular, for chronological age a linear increase emerged at 14 years of age, followed by a quite stable performance in later age (see Figure 2). The same was true when variations in rotation angles were analyzed. In the case of the development level, the performance was quite stable until 5 years of age and there was an increase in performance in the following considered ages. 

When the developmental level was considered as a function of the degree of rotation, for 0°, 45°, and 90° the best model was linear (explaining 20%, 26%, and 15% of the variance, respectively). In the case of 135°, the segmented regression was a better fit for performance (explaining 14% of variance). The curve was substantially flat until 5 years, with the breakpoint showing an increased performance. Rotation in the GTP at 180° was described by a null model. The descriptive statistics highlighted a gradually low performance in rotation accuracy in the GTP items with the progression of degree of rotation. 

It should be noted that the variance explained when the developmental level was considered as predictor is higher (32%) in comparison to chronological age (17%). In both models, the effect of gender was not significant. 

Taking together both spatial visualization and mental rotation tasks, performance increased as a function of chronological age and developmental level, even if the latter seemed to better describe the changes in visuospatial abilities. However, differences emerged: the best representative model for spatial visualization was the linear one, whereas the segmented one better represented mental rotation. These results can be discussed in line with TD evidence as well that of the AD. 

Firstly, it is important to note that the results of this study suggest the possibility of considering spatial visualization and mental rotation as distinct factors, despite the fact that such abilities are generally included in the larger construct of visuospatial abilities [3]. 

Spatial visualization showed to follow a linear increase in line with results in TD studies [32], even with the difference that, in previous TD studies, a limited age range was considered, while a larger age range was considered in the present study. However, the developmental level in individuals with DS appeared to be more informative of the development of such ability, showing to follow a linear trend, as previously suggested [51]. In particular, measuring the development level with a visuospatial reasoning task predicted the linear performance in a visuospatial task [50]. 

In contrast, performance in mental rotation showed to follow a segmented increase both for chronological and development level. To our knowledge, only Carretti et al. [47] specifically considered both linear and segmented trajectory in visuospatial performance, and the results of the present study are partially in line with it. In fact, while Carretti et al. [47] found that the VSWM task performance was better represented at chronological age by the segmented model, the developmental level is better represented by the linear model. Here we found that both chronological age and development level were better expressed by a segmented model. Several reasons can concur to explain the different results, such as the measures: visuospatial processing in Carretti et al. [47] and complex cognitive abilities, such as mental rotation, in the present study. 

Nevertheless, the segmented model offers interesting insight on how mental rotation develops in individuals with DS. Indeed it is interesting that when individuals with DS reach a development level (in terms of visuospatial reasoning) of 5 to 6, they also reach the necessary cognitive level to carry out the task: from 5 years of age the performance progressively increases because they demonstrate their ability to rotate stimuli. This is, at least in part, consistent with the TD literature showing that from 5 to 6 years of age the extrinsic frame of reference is developed [38]; moreover, there is evidence that it can be developed even earlier [37]. The ability to manage object to object relation (allocentric frame), going beyond the self to object relation (egocentric frame), can be very useful to approach this task. Indeed, there is evidence that strategies based on global rotation (i.e., mental rotation of the object as a whole, considering the rotation of objects independently from a personal point of view), are associated with good MR task performance [61]. Therefore, the extrinsic frame appears to be much more informative than that of chronological age when describing the developmental level that individuals with AD should reach in order to approach such stimuli. It should be noted that individuals with DS prefer to manage spatial information according the egocentric modality (in which the point of view of the individual is privileged, by using left and right [55,62]). It is possible that the use of an egocentric strategy (as in detection of left and right parts of the image) may help people with DS who approach the MR task. However, as the rotation degree increases (such as 180°, i.e., completely reversed), it becomes more difficult to manage the stimulus in an egocentric way, since the left and right are switched. This could be a plausible interpretation, however, it deserves to be studied in depth.

The analysis of the degree of rotation specified that the developmental level at 5–6 years was useful to approach the degree of rotation of a certain complexity, such as 135°. In fact, when the degrees of rotation were easier (45°, and 90°) the performance linearly increased with the developmental level. When individuals with DS reached 5–6 years of development level, they were able to approach the 135° degree. For larger degrees, like 180°, the developmental level was not sufficient; in this case performance was at chance level (as previously showed by Meneghetti et al. [26]). 

It should be noted that gender had no significant effect. The null effect of gender in the mental rotation ability contrasted with previous studies [41]. However, there is evidence showing that gender differences vary depending on age, from being small in children to medium in adults [40], and gender difference can be related to the type of stimuli used [43], as in our study based on the use of ghost images. At the same time, other studies examining visuospatial abilities in individuals with DS did not consider the role of gender [26,53]. This issue should be considered in further studies. 

The analysis of the trajectories in spatial abilities is relevant for their relation with other aspects of cognition and for school learning, such as math abilities in TD [32,63,64] but also in AD [59]. For example, Van Herwegen et al. [65] analyzed developmental trajectories in mathematical abilities in relation to domain-general (supported by intelligence and visuospatial abilities) and domain-specific abilities in math (non-symbolic and symbolic number abilities). Developmental trajectories were used, showing a general delayed performance in mathematical skills for DS and WS participants. In addition, similarities between DS and TD emerged since visuospatial skills predicted mathematical performance in both groups, on the contrary, domain-general abilities predicted performance in WS participants. Furthermore, the evidence that mental rotation trainings effectively improve math performance in TD children [66,67] offers insight on the impact that developmental profiles/trajectories have in describing cognitive skills (such as math abilities) and the necessity to train these skills in individuals with DS as well.

Although the results offer new and interesting insights, some limitations should be acknowledged. The main limitation of the study consists in the fact that the reported data are cross-sectional and not longitudinal, which prevents a more detailed account of the precise development of the two considered visuospatial abilities. Future studies assessing visuospatial abilities longitudinally should be conducted in order to better describe developmental trajectories. Another limit of this study is that it does not have a control group of TD children that would have allowed a direct comparison of trajectories with TD as suggested by Thomas et al. [10]. However, we should point out that a TD control group would have allowed comparisons between trajectories only in relation to the developmental level but not to the chronological age, since TD children reach a ceiling effect in the considered tasks after the age of eight. A further limitation concerns the unequal distribution of participants throughout the age range. Indeed, a more equal distribution across age could offer more consistent evidence. Another point to mention as a limit is that we only considered two types of visuospatial abilities. Despite these two abilities being related to everyday tasks [4,5], further studies should examine other types of visuospatial abilities more systematically (like spatial perception [3]), in larger contexts as well (like navigation, [9]; reorientation tasks, [68]) and in other atypical populations (such as William syndrome, [53,68]), considering their impact on other cognitive abilities [29].

## 5. Conclusions

To conclude, this report contributes to the understanding of changes in the cognitive development of individuals with DS, in particular with reference to two visuospatial abilities: spatial visualization and mental rotation. Results showed different pathways depending on the performance being plotted against chronological age or developmental level. Both chronological age and developmental level concur to explain the performance of both types of tasks, where the latter seemed to have a major role. Specifically, spatial visualization seemed to follow a linear development while mental rotation a segmented level of development with a critical age of 5–6; starting from this level of development, there is the propulsion to better perform a mental rotation task. 

## Figures and Tables

**Table 1:** Total raw scores for CPM (Colored Progressive Matrices), PMA–K1 (Primary Mental Abilities) and GTP (Ghost Picture Test) within each gender and total sample.

	CPM			PMA-K1			GTP		
	M	F	TOT	M	F	TOT	M	F	TOT
Mean	15.17	16.20	15.59	5.65	6.55	6.07	12.60	13.10	12.83
SD	4.64	4.86	4.74	1.95	2.13	2.07	3.37	3.75	3.52
Max	23	23	23	10	11	11	21	21	21
Min	7	7	7	2	2	2	6	5	5

## Data Availability

The data presented in this study are available on request from the corresponding author.
